# Population Dynamics and Survival Strategies of Two Endangered Ungulates in a Low Water-Availability Site of the Maya Forest of Mexico

**DOI:** 10.3390/ani15091307

**Published:** 2025-04-30

**Authors:** Rafael Reyna-Hurtado, Jonathan O. Huerta-Rodríguez, Alan Duarte-Morales, Itzel Poot-Sarmiento, Lizzi Valeria Martínez-Martínez, Manuel Alejandro Jiménez-Sánchez

**Affiliations:** 1 Department of Biodiversity Conservation, El Colegio de la Frontera Sur, Campeche 24500, Mexico; jonathan.huerta@posgrado.ecosur.mx; 2 Centro de Estudios del Desarrollo Sustentable y Aprovechamiento de la Vida Silvestre, Universidad Autónoma de Campeche, Campeche 24500, Mexico; al059027@uacam.mx; 3 Facultad de Ciencias Químico Biológicas, Universidad Autónoma de Campeche, Campeche 24500, Mexico; al051903@uacam.mx; 4 Comisión Nacional de Areas Naturales Protegidas, Calakmul, Campeche 24640, Mexico; lizzi.martinezmartinez@fao.org; 5 Centro Universitario de Ciencias Biológicas y Agropecuarias, Universidad de Guadalajara, Zapopan 44600, Mexico; manuel.jsanchez@alumnos.udg.mx

**Keywords:** occupancy, ephemeral ponds, abundance, co-occurrence, species conservation

## Abstract

White-lipped peccary (*Tayassu pecari*) and Central American tapir (*Tapirus bairdii*) are two endangered ungulates that inhabit the Maya Forest in Southern Mexico. These species need water sources almost every day to fill their ecological and physiological needs. How have they survived in a landscape like the Calakmul Biosphere Reserve where the water is very scarce and temporal? We analyzed 10 years of data for both species, collected through the intensive use of camera traps located in 18 ephemeral ponds of the Mexican side of the Maya Forest. These data in combination with occasional data on individual movements of both species collected with radiotelemetry allowed us to describe changes in occupancy, abundance, and movements that show the different strategies these species must cope with during dry periods. The white-lipped peccary population passed through cycles and disappeared from periods of water scarcity, and later, they appeared and stayed close to a few sources of water while the tapir population remained constant and occupied almost all sources of water throughout the years. This contribution increases the ecological knowledge and survival strategies of two endangered tropical ungulates of Mesoamerican Forests that have been disappearing at alarming rates in other forests of the region.

## Simple Summary

In the Maya Forest of Southern Mexico, water is a scarce resource for wild animals. Nonetheless, two of the most important Neotropical ungulates have survived in this dry scenario for thousands of years. Therefore, our objective was to describe the effect of water scarcity in the population of white-lipped peccaries and tapirs in the largest natural reserve in Mexico, Calakmul Biosphere Reserve. We found that white lipped peccaries disappeared when water was too scarce, while tapirs remained in the few ponds with water. We conclude that white-lipped peccaries are sensitive to water availability, and since climate change is affecting the rainy seasons, this species might be in grave danger of extinction. Therefore, it is of great importance to preserve the few ephemeral ponds to help these and other species to survive, for they contribute to the dispersal of many plant species that are used by local people, and they also represent a source of food for some human communities.

## 1. Introduction

In tropical forests, wildlife has adapted to cope with the uncertainty of resource availability in several ways. For example, some animals move long distances to access resources (e.g., peccaries) [1], others stay close to the resources and minimize energy expenditure (deer [2]; howler monkeys [3]), while others switch group size and composition to adapt to resources availability (red colobus; [4]) or modify the range they move every day [5].

Wildlife living in Neotropical forests located in the northern part of its distribution faces particular conditions, such as water scarcity in the dry season. Located in Southern Mexico, and Northern Guatemala and Belize, the Maya Forest, with almost 30,000 km^2^, is the largest tropical forest of Mesoamerica and is composed of the Calakmul Biosphere Reserve in Mexico, the Maya Biosphere Reserve in Guatemala, and the Rio Bravo-Dos Milpas private reserve in Belize [6]. The Maya Forest is characterized by water scarcity due to the karstic conditions of the soil that percolate the rainwater and storage water in the underground system of caves and rivers, leaving few sites in the ground where accessible water remains. These few sites are locally called *aguadas* and they are important sources of water for wildlife and people during the dry season [6]. These *aguadas* have been studied before and it has been documented that they are key pieces of the landscape for the survival of endangered species of wildlife in the Maya Forest [7,8,9,10].

Baird’s tapir (tapir hereafter; *Tapirus bairdii*) and white-lipped peccary (WLP hereafter; *Tayassu pecari*) are two endangered ungulates that inhabit the Maya Forest. Tapir is a member of the Perissodactyla order of mammals and, therefore, a relative of rhinos and horses, that lives from Mexico to Colombia and has been classified as Endangered by the IUCN Red List (www.iucnredlist.org) because the populations have decreased due to habitat loss and illegal hunting pressure [11]. It is a solitary species that socializes only when mating and while newborns remain with their mother for some time [12]. It feeds mainly on herbaceous plants and when available, on fallen fruits of the forest soils [13]. WLP belongs to the Artiodactyla order and, as distant relative of pigs, it lives in groups and visits water sites to refresh, drink, and wallow on the muddy areas [14]. WLP was classified as Vulnerable by the IUCN Red List (www.iucnredlist.org) in all its distribution range but in Mesoamerica, it has disappeared from 87% of its distribution range [15] due to illegal hunting pressure and because they need a large amount of habitat to sustain viable populations [1,16]. WLP lives in large groups of more than 300 individuals that roam together looking for high energy food, such as the fallen fruits and nuts of the Neotropical forests [17].

It has been documented that both species depend heavily on water availability to survive in the semidry forest of the Calakmul Biosphere Reserve in Southern Mexico. Groups of WLP traveled up to 17 km to reach some specific *aguadas* where they drink first and then wallow on the mud until they are fully covered in mud, a strategy believed to reduce the heat and prevent insects bites [7]; individual tapirs have been ascribed to some *aguadas* for up to 10 years, visiting them very frequently ([8]; 2019), and even *aguadas* have been proposed as “social arenas” for tapirs where they socialize and collect information on other conspecifics [12].

However, water availability in *aguadas* is not constant and depends on the rain patterns, some *aguadas* have water for several years and then dry up for another set of years. Previous studies have documented that *aguadas* passed through a process of drying up from 2008 to 2019 where less than 20% of the *aguadas* store water in 2019 [9,18]. This caused a dramatic effect on tapirs, in which several of them were seeing that year (2019), along the roads or visiting villages looking for water; several of them were attacked by dogs or were hit by cars on the road, causing death to some of them [18]. In another case, WLP were observed starving in year (2006), in which the largest *aguada* dried up for the first time in at least 40 years and several members of the four herds that were monitored with radiotelemetry were observed to be dead [7].

The use of automated cameras (camera traps) is a technique that has improved in the last 20 years and has been an instrumental tool for studying cryptic wildlife like these species of the tropical forest [19]. Camera traps allow the researcher to investigate population dynamics, behavioral traits, species association, and even human poaching, or the level of disturbance [20]. The use of camera traps to obtain information on the population dynamics of wildlife is a rich technique that produces valuable information about species that are otherwise hidden to the human eye. In the Calakmul Biosphere Reserve, some of us have conducted a long-term monitoring program of wildlife visiting *aguadas* for more than 10 years (R. Reyna-Hurtado pers. obs.). This program of *aguadas* monitoring has produced valuable information on the population of tapirs and WLP for these years and in several sites within the protected area that allow us to respond to some key questions of the two species. For example: How do the two species cope with water scarcity? What are the ecological strategies that allow them to survive in such a landscape?

To study population changes and trends of wildlife species, biologists have used several indicators, such as the Relative Abundance Index, a standardized index that could be compared among several studies because it takes into consideration the sampling effort. However, RAI lacks considering the differences in detection probability that species may have due to environmental or human conditions. Therefore, recently, occupancy models have been widely used to study wildlife populations because occupancy models do take into consideration detection probability and produce an index of occupancy that can be related to environmental or human conditions on a given landscape ([19]). Occupancy models can also test if some species co-occur in sites or times and are efficient statistical models that allow researchers to discriminate or rank covariates according to the effect on the occupancy probability of the study species [21]. On the downside, occupancy models assume populations are closed and reduce valuable data to a detection (1) or non-detection record (0) for specific periods of time.

Despite some research on tapirs and WLP, it remains unknown how the two species behave and cope with the changes in water availability over long periods, like 10 years, or to what degree their populations are affected by these changes in water availability. Therefore, in this study, we raised the following four general questions:

1. How the population index of relative abundance and the occupancy of the two species change over the years and over the sites?
2. Are fluctuations of WLP and Tapir populations in phase with variations in water availability?
3. What are the pond visitation patterns of the two species over ten years and what is affecting these patterns?
4. Finally, what are the differences in activity patterns of the two species?

We aimed to respond to these questions using the database we have produced from 2014 to 2023 on the visitation rate of the two species to *aguadas* in the heart of the Calakmul Biosphere Reserve in Southern Mexico. We assume that the visitation rate of the two species is a direct reflection of the population in the area given the documented high degree of dependency of the two species to water sources [7,12,13,14]. We used an array of methods, such as dynamic occupancy models that allow the continuous testing of occupancy probabilities on time [22,23]; the use of RAI as a standard index that reflects the changes in population dynamics on time and sites better [24], and the use of circular statistical analyses to elucidate activity patterns [23]. The final aim is to understand how water availability has impacted the use of water sources of the two ungulate species in a well-preserved forest that is subjected to extreme conditions, such as water scarcity, and collect valuable information that can be applied to the elaboration of conservation plans for these endangered species of ungulates of the Neotropical forests.

## 2. Materials and Methods

### 2.1. Study Area

**Figure 1:**
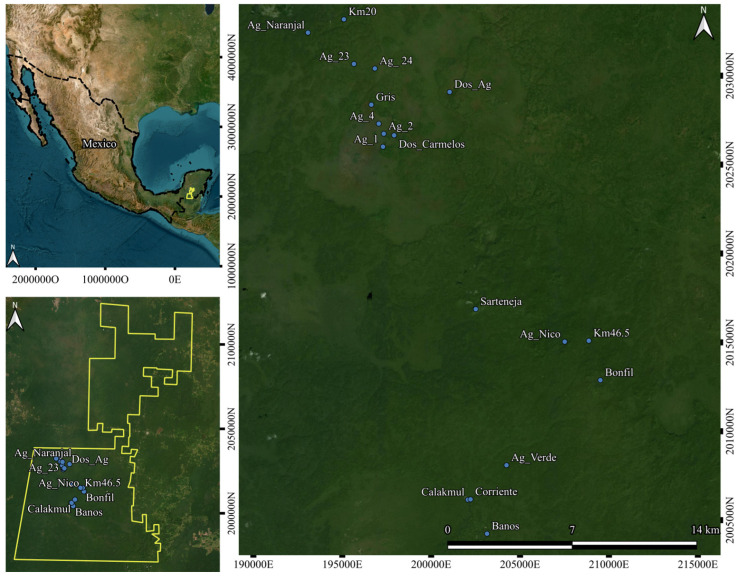
Calakmul Biosphere Reserve in Southern Mexico and camera trap positions within it.

Calakmul Biosphere Reserve (CBR hereafter; Figure 1), a protected area located in Southern Mexico, with an extension of 7289 km^2^, was decreed in 1989 in the municipality of Calakmul in the Southeast Campeche state, 19°15′17″ N, 90°10′89″ W [25]. CBR presents a tropical sub-humid climate, with rains in the summer (June to November) and dry months in winter–spring (February to May), having an annual mean temperature of 24.6 °C, and an annual mean precipitation of 1076.2 mm [26]. The reserve’s hydrography is determined by the amount of rainfall, but generally, the water flows underground and only in a few places, the water accumulates in the soil’s surface, forming ephemeral ponds [6]. These ponds (locally called *aguadas*) are essential components of the landscape for wildlife, as they are one of the few water sources of the region in the harsh months of the dry season [6,27]. In the CBR, the main vegetation types are the high evergreen forest, medium sub evergreen forest, low evergreen and deciduous forest, savanna, and hydrophytes [25,26].

### 2.2. Camera Trapping

Since February 2014 and up to July 2023, we conducted a long-term study focused on wildlife uses of ponds in the CBR, and conducted camera trapping surveys in 18 ponds (Figure 1). A single camera trap has been installed in each pond of a set of 10 to 18 ponds that were monitored during these years using the following brands: Reconyx PC800 Hyperfire professional (Reconyx, Inc., Holmen, WI, USA), Cuddeback (Cuddeback Inc., Green Bay, WI, USA) and Browning Strike Force (Browning Co., Spring, TX, USA). All these brands were similar in sensitivity, in detection range, and in shutter speed, and all of them used infrared rays at night. We kept the cameras in the same pond over the years even if some ponds dried up. The cameras were deployed in a tree base approximately 50 cm above the ground, and they were programmed to take pictures every time the sensor detected movement. The cameras were revised every two months to change memory cards and batteries.

### 2.3. Relative Abundance

To assure the independence of the data, for tapirs, all photographs obtained from the same species, but where individuals could not be recognized, were selected over a 60 min period, and we considered them as one event [28]. For WLP, we performed a similar approach, but instead of individual photos, we used group photos, and a group was defined by all the animals traveling or moving together in front of the camera in periods of less than 15 min (R. Reyna-Hurtado pers. obs.). To estimate the relative abundance index (RAI hereafter), the number of independent records of a selected species was divided by the number of days the cameras were deployed; then, the product was multiplied by 100 [24,29].

The RAI obtained was contrasted with water availability for the two species and it was also used to construct models to test the effect of three covariates. The three covariates used in the models were the following: (1) the Euclidian distance from the ponds to the single paved road that leads to the archeological zone; (2) the predominant type of vegetation of the pond (it was divided into low evergreen, medium sub evergreen forest, and medium deciduous forest), and (3) the degree of disturbance, which was measured as the frequency and number of people visiting the ponds, with the “medium” level being when the ponds were visited by the researchers of this study and additionally, by another research groups, or some tourists. It was considered a “low” level when they were only visited by our research group. We ran generalized linear models where RAI was the dependent variable, and the three covariates were the independent variables. We used the Akaike Information Criterion (AIC [30]) to select the most parsimonious model. We used R version 4. 4. 1 [31,32,33] with the package glm2 [34].

### 2.4. Occupancy

Following the methodology used by [35,36], we used the dynamic model of occupancy designed by [22] to calculate the probability of the occupancy, detection, colonization, and extinction of the two species in the ponds over the ten years of the camera-trapping monitoring period. As it was defined in [22], occupancy (*Ψ*) is the probability that a site is occupied by the study species and Detection (*p*) is the probability of detecting the study species, given presence ([22]). Colonization (γ) is the probability that an unoccupied site by the study species in a primary sampling period (t) is occupied in t + 1, and extinction (ϵ) is the probability that an occupied site by the study species in a primary sampling period (t) is unoccupied in t + 1 ([22]). The sampling units were the camera trapping sites (N:18) over the years 2014 to 2023, and to create a detection history per species, we pooled the records of detection (1) and non-detection (0) in 30 day intervals. For model selection, we used the Akaike’s information criterion (AIC; [30]). Models were performed using the Unmarked package in R v. 4. 4. 1 [37]. We follow a hierarchical approach to limit the number of models [38,39] where we individually create a model for each of the covariates to see their effect on the occupancy, detection, colonization, and extinction of the species by applying the AIC to select the most parsimonious one. Once we extracted the best covariate individually, we combined them in a multiple model where the best covariate affecting the occupancy, detection, colonization, and extinction for each species was performed. We used the same covariates described above for RAI and added the year as a linear factor.

To determine a possible relationship between the species’ occupancy and the presence of water, we used a multispecies model. Therefore, water presence was analyzed as another species in the Unmarked package in R v. 4. 4. 1 [37]. The model using multispecies allows estimating the probability of two or more species occurring simultaneously using a multivariate Bernoulli distribution as a function of covariates [40]. For the analysis, a detection pattern history was created for water as it was another species [36] and a null occupancy model was performed for the other two species separately, to visualize if there was any type of interaction between water and WLP and water and Tapir, and if it was significant. Additionally, we tested if there was any possible relationship between the presence/absence of the tapir and WLP with the presence/absence of the two large predators of the CBR, the jaguar (*Panthera onca*) and the puma (*Puma concolor*). We used the AIC weight to evaluate if there was co-occurrence among the water, or predators, and one of the two species of this study. The analysis was performed using the Unmarked package in R v. 4. 4. 1 [31,32,33].

### 2.5. Activity Patterns

Following the methodology used by [23,36], we also utilized the Kernel density estimation method to estimate the activity patterns of each species [31]. To perform the test, the packages Activity and Overlap were used in R v. 4. 4. 1 [31,32,33].

## 3. Results

Over the 10 years of camera trap monitoring on water ponds in the Calakmul Biosphere Reserve, we recorded 13,068 photos of tapir that were reduced to 834 independent records. In the same period, we obtained 34,376 photos of white-lipped peccaries that were also reduced to only 522 independent records.

### 3.1. Relative Abundance Index

We found a higher relative abundance index for tapir than WLP and it was consistent over all of the years and for almost all of the sites with a few exceptions (Table 1).

**Figure 2:**
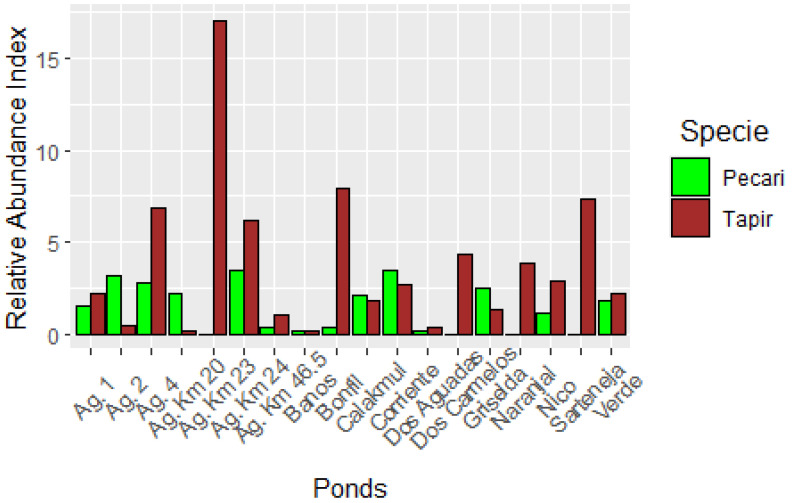
Relative Abundance Index (RAI) of *Tapirus bairdii* and *Tayassu pecari* during the period of 2014 to 2023 for 18 sites of the Calakmul Biosphere Reserve of Southern Mexico.

Tapir clearly favored some ponds, like Km 23, Bonfil, and Ag. 4, while WLP visited Km 24 and Corriente; however the difference among ponds was lower than for tapir (Figure 2).

**Figure 3:**
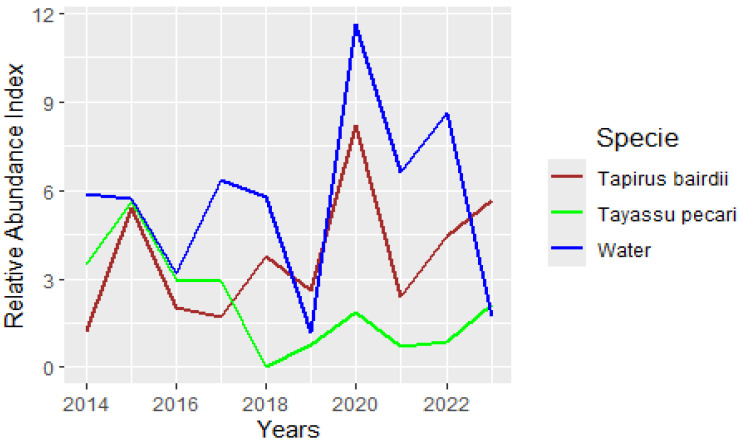
Relative Abundance Index (RAI) of *Tapirus bairdii* and *Tayassu pecari*, and water availability during the period of 2014 to 2023 on the Calakmul Biosphere Reserve of Southern Mexico.

One of the striking differences between the two species was the WLP’s total disappearance in all the sites for 2018, and a low RAI trend of this species from 2019 to 2023, while the tapir always visited the ponds reaching the highest index value for 2020 (Figure 3).

By running generalized linear models to test which variable best predicts the RAI estimated over the years for the two species, we found that tapir’s best predictor was the Distance to Roads, with a slightly negative relationship, while for WLP, the Perturbation Degree affected the RAI, which increases more in a low perturbation degree than in a medium perturbation degree. Nonetheless, these variables, as well as vegetation, or the combination of them, were not statistically significant for any of the two species (Table 2).

### 3.2. Occupancy Models

The dynamic occupancy models showed a consistently higher occupancy for both species, but a lower detection probability for WLP than for tapirs. They also showed a higher probability of extinction for WLP than tapirs, while the probability of colonization remained similar for both species. By running the models with the covariates, it turns out that the best model for tapir was the model where the probability of detection was affected by *Vegetation type* with a higher detection in the low dry forest than in both types of medium forests, where the Colonization rate was predicted by *Distance to Roads* in a positive relationship and extinction was negatively related to *Distance to Roads*. (Table 3). When running models with covariates affecting the parameters for WLP, it turns out that *Vegetation Type* influences the detection probability in a similar way to tapir (higher detection probability in the low dry forest and lower in both types of medium forests; Table 3). It turns out that this was the best overall model for WLP, while for tapir, it was also the best together with the additive model (Table 3).

### 3.3. Water and WLP and Tapir Relationships

**Figure 4:**
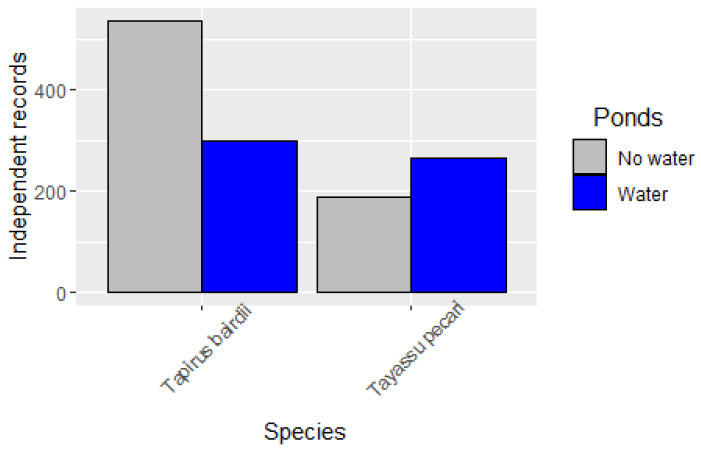
Visitation rate of ponds with and without water of independent records of *Tapirus bairdii* and *Tayassu pecari* during the period of 2014 to 2023 for 18 sites of the Calakmul Biosphere Reserve of Southern Mexico.

**Figure 5:**
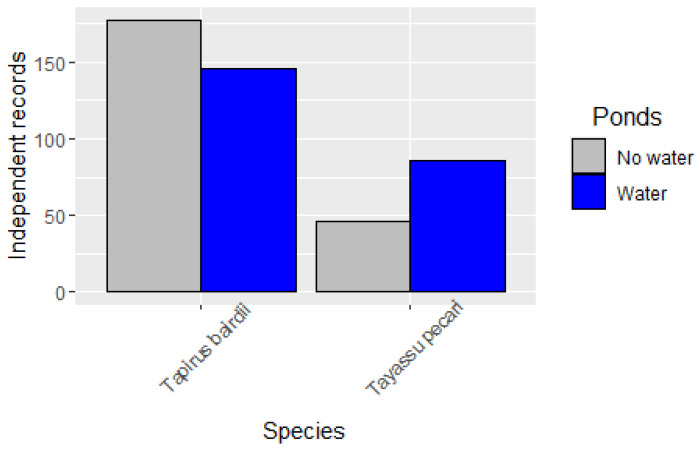
Visitation rate of ponds with and without water of independent records of *Tapirus bairdii* and *Tayassu pecari,* only during the dry seasons from the period of 2014 to 2023 for 18 sites of the Calakmul Biosphere Reserve of Southern Mexico.

The independent records of WLP over ten years showed that WLP is more actively visiting water filled ponds than dry ponds (Figure 4), and this pattern is clearer when we extracted the data only during the dry season months (February to May; Figure 5) for all years. On the other hand, tapirs visited more dry ponds than ponds with water over the years; however, the visitation rate of ponds with water also increased in the dry season (Figure 4 and Figure 5).

Using multispecies occupancy models, we also separately tested the co-occurrence of the tapir and the WLP with water availability over all the sites and years, and with the two large predators, jaguar (*Panthera onca*) and puma (*Puma concolor*). The best models for both species were using periods of 30 days and only for the dry season months (Table 4). We found that, using all the data, tapir and WLP show a co-occurrence with water in these sites and years, but when using only dry season data, only WLP favor the co-occurrence with water.

### 3.4. Activity Patterns

**Figure 6:**
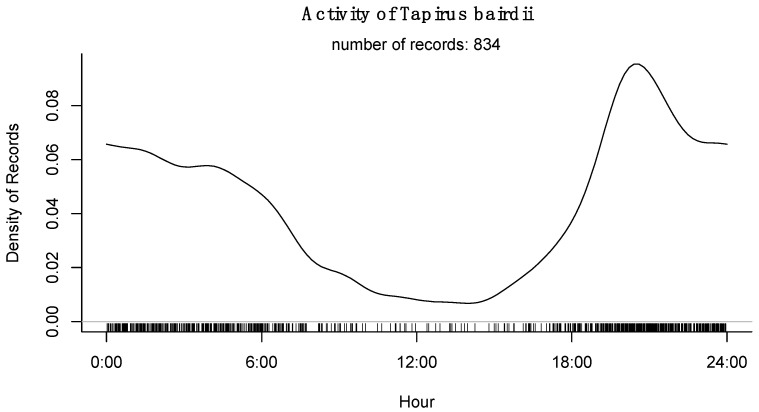
Activity patterns of *Tapirus bairdii* during the period of 2014 to 2023 for 18 sites of the Calakmul Biosphere Reserve of Southern Mexico (data taken with permission from [12]).

**Figure 7:**
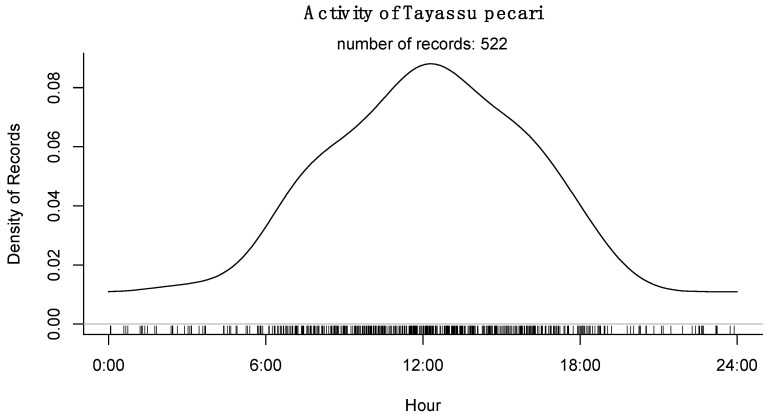
Activity patterns of *Tayassy pecari* during the period of 2014 to 2023 for 18 sites of the Calakmul Biosphere Reserve of Southern Mexico.

Finally, it was published recently [12], over the 10 years of the study period and the 18 study sites, tapirs showed a nocturnal pattern in general, with few records collected through daylight. There is a general preference for tapirs to visit ponds at early night hours (between 20:00 to 22:00 h; [12]), while WLP was clearly diurnal with a preference to visit ponds around midday (Figure 6 and Figure 7).

## 4. Discussion

This is the first study that presents a 10-year data period on the population of two endangered species of ungulates of the Maya Forest. Tapir and WLP were studied from 2014 to 2023 in 18 sites of the Calakmul Biosphere Reserve. The results showed that tapirs and WLP are important to the community of vertebrates and are frequent visitors of ponds. However, dynamic occupancy models showed that the WLP visitation rate was more episodic and less predictable than tapirs with a higher extinction rate and a lower colonization rate. WLP seems to appear and disappear from some periods of time; therefore, causing a lower detection probability but a higher occupancy probability according to the dynamic occupancy models. In WLP, detection probability was affected by vegetation type with detection being negatively affected in the sites on the medium sub evergreen forest and medium deciduous forest (Table 3). Tapir visitation rate was more consistent, and this species showed a higher detection probability but lower occupancy probability than WLP; the extinction rate was lower than WLP, but the colonization rate similar. Tapir detection in the best model was also affected negatively by *Vegetation Type* with lower estimates on the medium sub evergreen forest and medium deciduous forests than in the low dry forest, but in the additive models, *Distance to Roads* affected colonization rate in a positive way while extinction rate in a negative way. This model was among the best models for tapir (Table 3). However, important models for tapir have a better fit than for WLP after running a Chi Square Likelihood test with 5000 simulations (Table 3).

The results from occupancy models were consistent with the relative abundance index (RAI) that showed that WLP have a lower abundance than tapirs, and even totally disappeared in 2018, while tapirs always remained present and even increased their abundance to the highest peak in 2020. Tapir RAI seems affected negatively by the *Distance to Roads*, but positively affected for the Degree of Perturbation, with a slight increase in the areas with a medium level of perturbation. On the contrary, WLP RAI is affected negatively by the number of people visiting some of the ponds. These population patterns are according to what is known about the general behavior of these two species. WLP are known to have a less predictable pattern either spatially or temporally [16]. For example, in the Amazon forests, they appear and disappear from large areas and for periods of several years [41], with a cyclic pattern of more than 10 years to recover (or to travel back) the populations, or they disappear and appear without clear cycles [41]. These disappearances of WLP in several sites of their distribution range have been linked with diseases in some cases, with droughts in others, and with large movements of herds for other cases [41]. Tapirs, on the other hand, are known to show fidelity to specific sites and remain in relatively small areas throughout their life [12,42]. There are no reports of population cycles of the species over large geographical areas nor in time [13].

Contrary to what we expected, tapirs visited ponds without water more frequently than ponds with water, opposite to what we found for WLP. This behavior of tapirs visiting dry ponds could be a consequence of them looking for herbaceous species to feed on (as we could see in several photos of them). This behavior shows that tapirs use ponds in two ways: one to drink, refresh, and defecate in the water; and the other way, to feed on the herbaceous community of plants that are common on the edges of ponds, or in the forest gaps [12,13]. On the other hand, WLP showed a preference for visiting ponds with water, although they did also visit several ponds without water, maybe as an exploratory exercise. This behavior can be explained by the fact that they are actively looking for water almost on a daily basis to wallow, drink, and refresh themselves, and probably reduce the number of ectoparasites they may have [7,16,43]. The pattern of visiting ponds with water was more evident in the dry season for the WLP and, on a lower scale, it was also more evident for tapirs.

The co-occurrence models show that both species seem to have a co-occurrence with water using occupancy data. If we observed these results and the results from RAI analyses, we can conclude that WLP are more water-dependent than tapirs and that they are actively searching for water, especially during the dry season, while tapirs, on the other hand, can tolerate more periods without water and remain in the same sites in a more stable way than WLP, probably because one is a diurnal species (WLP) while the other is a clearly nocturnal species (tapir); this condition may help to reduce the heat. WLP is also known for traveling long distances looking for resources or water. In the same study area, it has been documented that herds of WLP can travel up to 17 km in one or two days looking for water [1]. These long trips can take the groups of WLP to areas that they may have not visited in several months or years [1,16]. While this behavior has not been reported for tapirs yet, and apparently tapirs have a more stable and smaller home range [8], there is empirical evidence that tapirs can also perform some trips to visit some specific sites, such as small ponds or other drinking sources (stones called locally *sartenejas*), and that these trips can be longer than what we have recorded before ([8]; R. Reyna-Hurtado unpublished data).

These differences in searching for or accessing resources could be consequences of the differences in social behavior of the two species; one (WLP) showing a high social behavior by forming large and cohesive groups that travel together for long distances within huge home ranges, and with a social mechanism in which all of the groups defend together [7], and the other one (tapir), being a solitary species most of the time (although they are social 13% of their time in the wild; [12] and living in more stable and smaller home ranges [8,42]; W. Martinez et al. unpublished).

Tapirs are nocturnal most of their time as it has been reported elsewhere [12,13,44] with a marked preference for early night hours, specifically from 18:00 to 24:00, with a peak between 20:00 to 22:00 h, while WLP are mostly diurnal species, with very few records in the night (only in a few very hot nights) and with a peak of pond visitation time at midday. These results are consistent with what we know previously, but 10 years and 18 sites demonstrated that both species are consistent in their ecological strategies of accessing resources, and that water resources are used in the same sites, but at different times. This is probably an evolutive–ecological strategy that both species have developed to assure access to water without the need of competing for it.

Both species also showed co-occurrence with jaguar and pumas; this may be true, or may be a consequence of ponds being sites that animals visit frequently to access water; the co-occurrence is a consequence of the value of the ponds for the survival of several species of wildlife. We have also collected evidence that tapirs visit ponds to access information from conspecifics and the ponds are becoming social arenas for the species [12,45]. So, the presence of predators and ungulates in a pond may be due to water needs, or to collect information on conspecifics, or to actively follow one species to another, or a combination of these. It is necessary to test this in the future with data at a fine temporal scale.

In previous studies, it has been demonstrated that ponds play a vital role in the survival of the two species. For example, [1] explicitly demonstrated that WLP depend heavily on ponds for survival during the dry season and the species even behaved as central place foragers with the ponds as the central place of the group’s movement during the dry season. Tapirs also depend on water ponds to refresh and drink, as has been demonstrated by [46] and [8,9,12]; however, ponds could be playing the role of “social arenas” for tapirs and apparently, some individuals may be visiting the ponds to find mates, or acquire information about other individuals of the area, such as males or estrous females ([12]; R. Reyna-Hurtado pers. obs.). Water source sites have been found to also be sites for socialization in Saiga antelope (*Saiga tatarica*; [45]). Recently, we found a high dynamic interaction for tapirs among two males and two females in one pond, where over five days, they fought, socialized, and probably mated (Reyna-Hurtado et al. 2025 [47]).

Here, we found that tapirs and WLP perform different behaviors that are probably different ecological strategies to cope with this uncertainty of water availability, typical of the Calakmul Biosphere Reserve. On one hand, tapirs remain frequent visitors of ponds and also visit many dry ponds; they do that with a relatively high occupancy probability and a high (and somehow) stable relative abundance index. On the other hand, WLP show large changes in the population parameters that reflect on a low detection probability and a large variation in the relative abundance index, that at least for one year, went down to zero (disappeared). There is always the question of what happens with the groups of WLP that year (2018); there is evidence of mortality among some of the groups (R. Reyna-Hurtado and N. Arias Dominguez pers. obs.), but the possibility of them traveling far away from the study area is a real one, as it happened between the 2005 and 2006 dry seasons when some groups that were radiocollared left the study area and returned months later [7].

These two ecological strategies of the two more endangered ungulates of the Maya Forest are interesting, as these are probably the key factors that allow them to survive in this water-scarce forest. Tapirs remain close to the few ponds that have water and frequently visited other ponds to feed on and to gather information on water availability, while WLP travel larger distances, and when finding water, remained close to the few ponds that had water that season. These results point out the importance of the conservation of the whole set of ponds and the whole landscape. Conserving these two species that depend on ponds and large amounts of well-conserved forests is a challenge, but the Calakmul Biosphere Reserve is probably one of the few places in Mesoamerica where this can be achieved, as its extension and position adjacent to the Maya Biosphere Reserve in Guatemala form a large landscape suitable for populations of animals that need large extensions of habitat to sustain viable populations.

We must preserve the forest and the system of ponds to allow tapirs and WLP to perform their natural behavior and their ecological strategies that have been developed in the area to secure access to water in this limited landscape. We must preserve the two species and their behaviors by allowing them free access to all ponds, by assuring the populations are not fragmented due to roads, railroads, and other infrastructure projects and by protecting them from hunters across the whole region. Conserving the two large and endangered ungulates of the Maya Forest will indicate that the forest is in its best state and that effective protection is taking place in the area. More research will be needed to understand if individuals of the two species move using spatial memory, and if they use ponds for other purposes, such as social arenas. As forest fragmentation is advancing in all Mesoamerica forests, it is imperative to conserve the Maya Forest and the two endangered ungulates and allow them to perform their ecological strategies that allow them to survive in the area and fill their ecological role.

## 5. Conclusions

This study advanced in the understanding of the population dynamics of two endangered ungulates in a preserved tropical forest. Tropical forests in good conditions of conservation are disappearing at alarming rates and the two species face diverse challenges to survive in other human-impacted forests; therefore, studying them in a well conserved forest and with particular conditions, such as water scarcity, allowed us to understand the ecological strategies that both species perform to survive in the area, strategies that probably have been developed in evolutive time and are the product of the interaction among species, water, and forests over many years.

Understanding these ecological strategies may be useful in another context, when efforts to save populations of these species in fragmented forests, or if climate change intensifies and less water is available in the area, as it has been predicted [48] and shown for water ponds in the area already [9]. Finally, this study can also be useful for understanding the dynamics of ungulate species adapted to dry or semidry tropical forests and how they cope with water scarcity.

## Figures and Tables

**Table 1:** Relative Abundance Index (RAI) and Standard Error (SE) of all sites and years for *Tapirus bairdii* and *Tayassu pecari* in ponds of the Calakmul Biosphere Reserve of Southern Mexico.

Specie	RAI of Total Sampling Time and Sites
*Tapirus bairdii*	2.49 (0.77)
*Tayassu pecari*	1.56 (1.62)

**Table 2:** Models affecting the Relative Abundance Index (RAI) for *Tapirus bairdii* and *Tayassu pecari* using three covariables to test for their effect in ponds of the Calakmul Biosphere Reserve of Southern Mexico (the best models are highlighted).

Species	Models Affecting RAI	AIC	Adjusted R^2^	Model *p* Value
***Tapirus bairdi***	**RAI** **~Distance to Roads + Vegetation Type + Perturbation Degree**	**107.5**	**0.13**	**0.34**
	RAI~Distance to Roads + Vegetation Type	110.2	0.03	0.51
	**RAI** **~Distance to Roads**	**106.3**	**0.00**	**0.32**
	RAI~Vegetation Type	109.4	0.01	0.48
	RAI~Perturbation Degree	109.2	0.11	0.87
	RAI~Distance to Roads + Perturbation Degree	109.8	0.10	0.71
	**RAI** **~Vegetation Type + Perturbation Degree**	**107.3**	**0.13**	**0.31**
***Tayassu pecari***	**Models affecting RAI**	**AIC**	**Adjusted R^2^**	***p* value**
	RAI~Distance to Roads + Vegetation Type + Perturbation Degree	69.31	−0.07	0.57
	RAI~Distance to Roads + Vegetation Type	69.00	−0.07	0.59
	**RAI** **~Distance to Roads**	**65.43**	−**0.05**	**0.77**
	RAI~Vegetation Type	67.06	0.01	0.43
	**RAI** **~Perturbation Degree**	**64.46**	**0.04**	**0.27**
	**RAI** **~Distance to Roads + Perturbation Degree**	**65.23**	**0.04**	**0.32**
	RAI~Vegetation Type + Perturbation Degree	67.35	0.03	0.43

**Table 3:** Dynamic Occupancy Models for *Tapirus bairdii* and *Tayassu pecari* using three covariates to test for their effect on Occupancy *Psi* (*Ψ*), Detection probability *Det* (*p*), Colonization *Col* (***γ***), and Extinction *Ext* (*ϵ*) in ponds of the Calakmul Biosphere Reserve of Southern Mexico (the best models are highlighted).

Species	Models	AIC	Estimate and Prob. of Occu. *Psi* (*Ψ*)	Estimate and Prob. of *Det* (*p*)	Estimate and Prob. of *Col* (*γ*)	Estimate and Prob. of *Ext* (*ϵ*)	Model *p* Value
***Tapirus bairdi***	Null Model	814.27	1.97 (0.878)	−0.51 (0.377)	0.54 (0.632)	−1.58 (0.170)	0.00
	**Det (*p*)** **~Vegetation Type**	**795.67**	**1.96 (0.877)**	**0.87 (0.705)**	**0.66 (0.66)**	**−1.73 (0.151)**	**0.14**
	Det (*p*)~Perturbation Degree	799.33	2.0 (0.880)	-0.39 (0.402)	0.781 (0.686)	−1.72 (0.152)	0.06
	**Psi (** ***Ψ*)** **~Null** **Col (*γ*)** **~Distance to Roads** **Ext (** ***ϵ*)** **~Distance to Roads** **Det (*p*)** **~Vegetation Type**	**795.05**	**1.87 (0.867)**	**0.87 (0.705)**	**1.11 (0.752)**	**−1.63 (0.162)**	**0.14**
	Psi (*Ψ*)~Null Col (*γ*)~Distance to Roads Ext (*ϵ*)~Distance to Roads Det (*p*)~Perturbation	799.53	1.93 (0.874)	−0.43 (0.392)	-1.43 (0.744)	0.52 (0.164)	0.07
***Tayassu pecari***	Null model	576.59	6.43 (0.998)	−1.36 (0.205)	0.53 (0.631)	−1.03 (0.236)	0.00
	**Det (*p*)** **~Vegetation Type**	**557.3**	**8.28 (1.00)**	**−0.07 (0.482)**	**1.29 (0.784)**	**−1.13 (0.244)**	**0.01**
	Det (*p*)~Perturbation Degree	559.92	7.52 (0.99)	−2.65 (0.06)	5.69 (0.997)	−1.25 (0.222)	0.01
	Psi (*Ψ*)~Null Col (*γ*)~Perturbation Degree Ext (*ϵ*)~Perturbation Degree Det (*p*)~Vegetation Type	562.68	8.47 (1.00)	−0.07 (0.481)	−0.04 (0.489)	−0.19 (0.451)	0.00

**Table 4:** Occupancy models for *Tapirus bairdii* and *Tayassu pecari* by years using *Panthera onca*, *Puma concolor,* and water availability to test for their co-occurrence in ponds of the Calakmul Biosphere Reserve of Southern Mexico.

Species and Water Co-Occurring	AIC	AIC Weights	Occupancy Estimates (in Logit Scale) and *p* Values (in Brackets)
*Tapirus bairdi* and water (only dry seasons)			
Co-occurrence	297.19	0.83	0.89 (0.02)
No Co-occurrence	300.38	0.17	
*Tapirus bairdi* and water (all year)			
Co-occurrence	3027.47	0.80	2.17 (0.00)
No Co-occurrence	3030.42	0.19	
*Tapirus bairdi* and *Panthera onca*			
Co-occurrence	2939.54	0.80	2.14 (0.00)
No Co-occurrence	2942.32	0.20	
*Tapirus bairdi* and *Puma concolor*			
Co-occurrence	3301.05	0.83	2.21 (0.00)
No Co-occurrence	3304.21	0.17	
*Tayassu pecari* and water (only dry seasons)			
Co-occurrence	234.74	0.57	1.46 (0.00)
No Co-occurrence	235.33	0.43	
*Tayassu pecari* and water (all year)			
Co-occurrence	2464.00	0.78	2.09 (0.00)
No Co-occurrence	2466.53	0.22	
*Tayassu pecari* and *Panthera onca*			
Co-occurrence	2376.02	0.77	2.07 (0.00)
No Co-occurrence	2378.43	0.23	
*Tayassu pecari* and *Puma concolor*			
Co-occurrence	2737.65	0.79	2.13 (0.00)
No Co-occurrence	2740.32	0.21	

## Data Availability

The data sets presented in this article are not readily available because the data are part of an ongoing study.
